# Correlation of CCL20 expression in rectal mucosa with the development of ulcerative colitis-associated neoplasia

**DOI:** 10.3892/ol.2013.1528

**Published:** 2013-08-16

**Authors:** KIYOSHI HASHIMOTO, SUSUMU SAIGUSA, TOSHIMITSU ARAKI, KOJI TANAKA, YOSHIKI OKITA, HIROYUKI FUJIKAWA, MIKIO KAWAMURA, YOSHINAGA OKUGAWA, YUJI TOIYAMA, YASUHIRO INOUE, KEIICHI UCHIDA, YASUHIKO MOHRI, MASATO KUSUNOKI

**Affiliations:** 1 Department of Gastrointestinal and Pediatric Surgery, Division of Reparative Medicine, Institute of Life Sciences, Mie University Graduate School of Medicine, Tsu, Mie 514-8507, Japan; 2 Department of Innovative Surgery, Division of Reparative Medicine, Institute of Life Sciences, Mie University Graduate School of Medicine, Tsu, Mie 514-8507, Japan

**Keywords:** ulcerative colitis, CCL20, CCR6, field effect, surveillance

## Abstract

Chronic inflammation increases the risk of developing several gastrointestinal malignancies. Chemokines that are produced by colonic epithelial cells play significant roles in the maintenance and repair of the epithelial barrier. The present study aimed to clarify whether the expression of CCL20 and its receptor, CCR6, was correlated with the development of ulcerative colitis (UC)-associated neoplasia. A total of 93 patients with UC who underwent proctocolectomies were enrolled in the present study. Immunohistochemical analysis for CCL20 and CCR6 expression in the rectal mucosa was performed and the correlation between expression and the pathogenesis of UC-associated neoplasia was investigated. A total of 16 (17.2%) patients presented with UC-associated neoplasia. The immunohistochemistry (IHC) score for CCL20 was significantly increased in the patients with a mild form of the disease (P=0.0363). The IHC score for CCL20 expression in the patients with UC-associated neoplasia was higher compared with the patients without neoplasia (P=0.0294). In contrast, there was no significant correlation between CCR6 expression and the clinicopathological variables. The logistic regression analysis revealed that a high IHC score for CCL20 expression in the rectal mucosa and a disease duration of more than eight years were significantly correlated with the development of UC-associated neoplasia (P<0.05). The results suggest that an evaluation of CCL20 expression in the rectal mucosa may be useful to identify patients who are at a high risk for developing UC-associated neoplasia. However, a selection bias existed in the present study due to the fact that the patient population that was enrolled was not representative of a typical surveillance patient population.

## Introduction

Inflammatory bowel disease (IBD) is characterized by a chronic inflammation of the gastrointestinal tract. Ulcerative colitis (UC) is the most common form of IBD and patients with UC are predisposed to developing colorectal cancer. A longer duration of disease and a greater extent of colitis, i.e. pan- or left-sided colitis, are believed to increase the risk of developing UC-associated cancer (UCAC) (1). Studies have shown that the risk of developing colorectal cancer in UC patients is 2, 8 and 18% following 10, 20 and 30 years of active disease, respectively (2). Despite epidemiological and experimental evidence demonstrating the increased risk of developing UCAC, the mechanisms of neoplastic transformation and progression remain unclear. The development of UCAC is believed to arise from widespread alterations that are caused by a combination of genetic and epigenetic factors, in addition to host and microbial affects. UCAC is occasionally referred to as an ‘inflammation dysplasia carcinoma sequence’, which differs from sporadic colon cancer (3,4). Furthermore, previous studies have suggested that the ‘field effect’, in which genetic and molecular alterations that are caused by chronic inflammation are identified in neoplastic lesions and non-neoplastic epithelia, is common in epithelial carcinogenesis (5,6).

Chemokines that are produced by colonic epithelial cells play significant roles in the maintenance and repair of the epithelial barrier and in cancer progression (7,8). CCL20, also known as macrophage inflammatory protein (MIP) 3α or liver and activation regulated chemokine, is predominantly expressed in the inflamed intestinal epithelium and plays a significant role in lymphocyte and dendritic cell activation and recruitment to the colonic epithelium (9,10). Previous studies have demonstrated that CCL20 expression levels in the colonic epithelia of patients with IBD were higher than in the normal colonic epithelia (11,12). Furthermore, neutralization of CCL20 expression using its monoclonal antibody has been shown to reduce 2,4,6-trinitrobenzen sulfonic acid (TNBS)-mediated colonic injury and T-cell recruitment (13). CCR6 is a functional receptor for CCL20 that is expressed in lymphocytes, immature dendritic cells, activated neutrophils and lymphoid tissues, including the lymph node, spleen and appendix (9,14). In addition, Varona *et al*(15) conducted an *in vivo* study demonstrating that CCR6 plays a crucial role in the development of IBD. These findings suggest that the CCL20/CCR6 axis may contribute to chronic inflammation of the colonic mucosa.

The present study investigated whether an evaluation of CCL20 and CCR6 expression in the rectal mucosa would be useful for predicting the development of UC-associated neoplasia.

## Materials and methods

### Patients and samples

A total of 93 formalin-fixed, paraffin-embedded (FFPE) tissue samples were obtained from patients with UC who underwent proctocolectomies between 2003 and 2011 in Mie University Hospital (Tsu, Mie, Japan). The patients with right-sided or segmental colitis and proctitis, acute fulminating, first attack-type disease or those that were <15 or >60 years old at the onset of the disease were excluded from the study. Approval for this study was obtained from the ethics review board of Mie University Hospital. All the patients provided written informed consent to allow the collection and use of their tissues for the present study.

### Immunohistochemistry (IHC)

The FFPE specimens were sliced into 2-μm thick sections. Following deparaffinization and dehydration, the sections were incubated in 10 mM sodium citrate buffer (pH 6.0) and autoclaved at 121°C for 10 min for antigen retrieval. Following an additional incubation in 3% hydrogen peroxide for 10 min, the sections were blocked and incubated with a primary antibody overnight at 4°C. Human CCL20/MIP-3α antibodies (monoclonal mouse IgG_1_ clone no. 67310; dilution, 1:250; R&D Systems, Minneapolis, MN, USA) and human CCR6 antibodies (monoclonal mouse IgG_2B_ clone no. 53103; dilution, 1:50; R&D Systems) were used as the primary antibodies for the implementation of the labeled streptavidin-biotin method using the Envision™+ Dual Link System-horseradish peroxidase (HRP) and 3,3′-diaminobenzidine (DakoCytomation, Glostrup, Denmark) staining. All the sections were counterstained with hematoxylin, then dehydrated and mounted. A minimum of three sections/specimen were stained to confirm reproducibility. Negative controls were run simultaneously using pre-immune immunoglobulin.

### Immunohistochemical evaluation

The sections were observed under a light microscope. The IHC scores were calculated by multiplying the percentage of the positive epithelial cells (0–100%) by the staining intensity, as described in a previous study (16). The staining intensity was scored as follows: 0, negative; 1, weak; 2, moderate; and 3, strong. The IHC score ranged from 0–300. Each sample was scored in a blinded manner by two investigators who did not have any clinical or pathological information with regard to the origin of the samples.

### Statistical analysis

All statistical analyses were performed using Stat View 5.0 for Windows (SAS Institute Inc., Cary, NC, USA). The contingency tables were analyzed using Fisher’s exact probability test or the χ^2^ test with Yates’ correction. The correlation between the continuous and categorical variables was evaluated using the Mann-Whitney U test for two groups and the Kruskal-Wallis test for more than three groups. A non-parametric receiver operating characteristic (ROC) analysis was performed to calculate the best cut-off value for each IHC score that was predictive of the development of UC-associated neoplasia, using MedCalc 7.2 for Windows (MedCalc, Mariakerke, Belgium). The logistic regression analysis was used to evaluate whether CCL20 and CCR6 expression in the rectal mucosa predicted the development of UC-associated neoplasia. P<0.05 was considered to indicate a statistically significant difference.

## Results

### Patient demographics and disease characteristics

The characteristics of the patients are shown in Table I. The median age at UC diagnosis was 29 years (range, 17–59 years). A total of 74 patients presented with pancolitis and the remainder presented with left-sided colitis. With regard to the degree of inflammation, 46, 39 and 8 patients demonstrated mild, moderate and severe inflammation, respectively. A total of 16 patients (17.2%) had UC-associated neoplasia, seven of whom developed UCAC with dysplastic lesions and nine who had only dysplastic lesions. The median disease duration was six years in the patients with non-neoplasia, eight years in the patients with dysplasia and 19 years in the patients with UCAC.

### Immunohistochemical findings and evaluation of CCL20 and CCR6 expression

**Figure 1:**
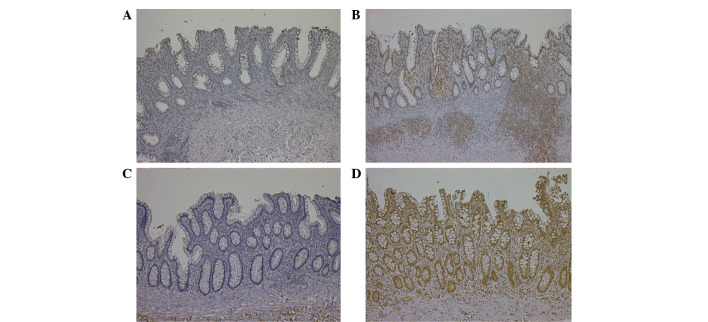
Immunohistochemical findings for CCL20 and CCR6 in the rectal mucosa. CCL20 expression was observed in the nuclei of the epithelial and inflammatory cells and the lymphoid follicles. (A) Weak and (B) strong expression of CCL20 in the rectal mucosa. CCR6 expression was observed in the nuclei and cytoplasm of the epithelial, infiltrating inflammatory and endothelial cells. (C) Weak and (D) strong expression of CCR6 in the rectal mucosa (labeled streptavidin-biotin staining; magnification, ×100).

CCL20 expression was observed in the nuclei of the epithelial cells, the inflammatory cells and the lymphoid follicles (Fig. 1A and B). CCR6 expression was observed in the nuclei or cytoplasm of the epithelial cells, the infiltrating inflammatory cells and the endothelial cells (Fig. 1C and D). The median IHC scores of CCL20 and CCR6 were recorded as 20 (range, 0–285) and 40 (range, 0–270), respectively.

### Correlation of CCL20 and CCR6 expression in the rectal mucosa with clinical outcome

**Figure 2:**
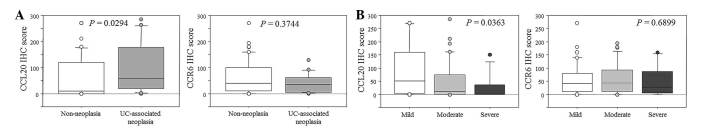
(A) Correlation of epithelial CCL20 and CCR6 IHC score with the development of UC-associated neoplasia. The CCL20 IHC score of the UC-associated neoplasia group was higher than that of the non-neoplasia group (P=0.0294; Mann-Whitney U test). (B) Correlation of the epithelial CCL20 and CCR6 IHC scores with disease severity. The epithelial CCL20 expression in the patients with a mild or moderate form of the disease was elevated compared with those with severe disease (P=0.0363; Kruskal-Wallis test). IHC, immunohistochemistry; UC, ulcerative colitis.

The patients with low- or high-grade dysplasia and UCAC were classified as the UC-associated neoplasia group and the remainder were classified as the non-neoplasia group. There were no significant differences in the gender, extent of disease or degree of inflammation between the non-neoplasia and UC-associated neoplasia groups. The patients with UC-associated neoplasia had a significantly longer disease duration than those without neoplasia (11 vs. six years, respectively; P=0.0172; Table II). The IHC score for CCL20 in the UC-associated neoplasia group was higher than in the non-neoplasia group (P=0.0294). In contrast, there was no significant correlation in CCR6 expression between the non-neoplasia and UC-associated neoplasia groups (P=0.3744; Fig. 2A). The IHC score for CCL20, but not CCR6, was significantly increased in the patients with a mild form of the disease (P=0.0363; Fig. 2B). The ROC analysis determined that the optimal cut-off values for the IHC score of CCL20 and CCR6 were 20 and 92, respectively. In the logistic regression analysis, the duration of the disease (>8 years) (2,17,18) and the IHC scores of CCL20 above the cut-off value were significantly associated with the development of UC-associated neoplasia (P=0.0287; Table III).

### Comparison of CCL20 and CCR6 expression in UCAC and sporadic cancer

**Figure 3:**
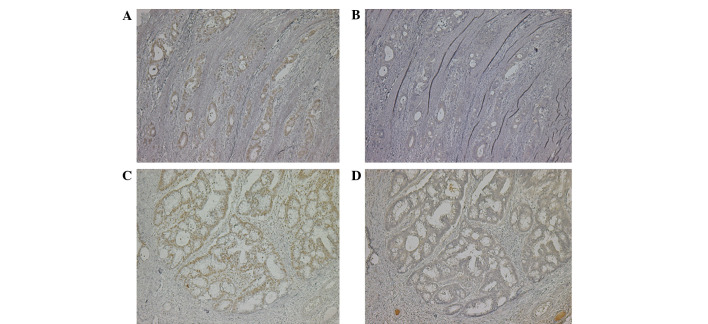
CCL20 and CCR6 expression in UC-associated cancer (UCAC) and sporadic cancer. (A) CCL20 and (B) CCR6 expression in UCAC. (C) CCL20 and (D) CCR6 expression in sporadic colon cancer. CCL20 expression was observed in the nuclei of UCAC and sporadic colon cancer cells. CCR6 expression was observed in the cytoplasm of the cancer cells (labeled streptavidin-biotin staining; magnification, ×100). UC, ulcerative colitis.

Next, the expression of the two markers, CCL20 and CCR6, were compared in UCAC and sporadic colon cancer. Fig. 3 shows the immunohistochemical results for CCL20 and CCR6 in UC-associated and sporadic colon cancer. CCL20 expression was observed in the nuclei of the UC-associated and sporadic colon cancer cells. In contrast, CCR6 expression was observed in the cytoplasm of the cancer cells. The expression of CCR6, but not CCL20, was increased in UCAC compared with sporadic colon cancer (P=0.0316; Table IV).

## Discussion

Current surveillance guidelines recommend that a colonoscopy with random biopsies should be collected at 10-cm increments along the colonic mucosa or that target biopsy testing should be conducted every 1–2 years for chronic UC (17,19,20). However, this type of surveillance has demonstrated several limitations, including sampling errors and difficulties in the macroscopic diagnosis of neoplastic lesions that are flat or diffusely infiltrative (21,22). Novel optical techniques, including chromoendoscopy, narrow band imaging, confocal laser endoscopy and partial-wave spectroscopic microscopy, have been reported to aid in the detection and diagnosis of neoplastic lesions in patients with UC (23–26). Further reliable and effective diagnostic approaches are urgently required to improve the surveillance of UCAC.

In the present study, a high expression level of CCL20 and a low expression level of CCR6 in the rectal mucosa were correlated with the development of UC-associated neoplasia. These results suggested that an evaluation of CCL20/CCR6 expression in the rectal mucosa may be useful in the identification of patients who are at high risk for developing UC-associated neoplasia.

Several studies have demonstrated that CCL20 expression is increased under inflammatory conditions and that CCR6 expression, while not affected by inflammation, is upregulated during epithelial differentiation (10,27,28). In the present study, CCR6 expression was not observed to be correlated with the severity of UC, which was similar to the results of previous studies, and CCL20 expression was shown to be affected by the severity of UC. Notably, the CCL20 expression in the epithelial cells of patients with mild or moderate disease forms was elevated compared with patients with severe disease. This suggested that the patients with a good control of the mucosal inflammation or repeated active and remission by conventional treatments for UC may have a high level of CCL20 expression.

Cook *et al*(14) reported that CCL20 and CCR6 were separately expressed by adjacent cell populations within the Peyer’s patch, suggesting that CCL20 may have paracrine functions. In contrast, the co-expression of CCL20 and CCR6 in the same cells suggested that a CCL20/CCR6 interaction mediated the autocrine or paracrine effects on intestinal epithelial cells (27). However, there was no significant correlation between CCL20 and CCR6 expression in the rectal mucosa in the present study. This may be explained by the presence of multiple receptors for CCL20 (data not shown). The percentage of rectal epithelial cells co-expressing CCL20 and CCR6 was 20.4% of all the patients, including 23.4% (18/77) of patients without neoplasia and 6.3% (1/16) of patients with UC-associated neoplasia. Thus, the present data suggested that CCL20/CCR6 signaling may have various physiological functions and that these proteins may exhibit differential interactions in the context of UC compared with non-UC.

In sporadic colorectal cancer, CCL20/CCR6 signaling has been shown to play a role in the proliferation and migration of colorectal cancer cells and in the promotion of liver metastasis via the autocrine and paracrine functions (27,29). Several studies have suggested that the prognoses of UCAC and sporadic colon cancer are similar (30–32). However, this remains a controversial topic. Furthermore, Mikami *et al*(33) reported that poorly-differentiated UCAC with invasion of the submucosa or with a greater dependence on CD44 cleavage may influence a poor prognosis. In the present study, CCR6 expression was observed to be more frequent in UCAC compared with sporadic colon cancer. The results suggested that CCR6 expression may be associated with the malignant potential of UCAC. However, it should be noted that the data did not demonstrate whether CCL20/CCR6 signaling played a role in the carcinogenesis of UC. In future studies, this possibility may be investigated using transcriptional and *in vivo* studies.

In conclusion, the results of the present study suggested that an evaluation of CCL20/CCR6 expression in the rectal mucosa may be useful for the identification of high-risk patients with UC-associated neoplasia. However, the data in this study should be interpreted with caution. Firstly, a selection bias was present due to the fact that the patient population that was enrolled in the study was not representative of a typical surveillance patient population. Furthermore, this study included a small number of UC patients. Hence, further research, including a prospective and multicenter study using a collection of biopsy samples from patients with UC, is required to assess the utility of these markers for routine clinical use.

## Figures and Tables

**Table I:** Patient characteristics (n=93).

A, Characteristics	Value
Gender, n	
Male	49
Female	44
Age at UC diagnosis, years (range)	29 (17–59)
Extent of disease, n	
Pancolitis	74
Left-sided colitis	19
Duration of disease, years (range)	7 (1–28)
Degree of inflammation, n	
Mild	46
Moderate	39
Severe	8
Neoplasia classification, n	
Without neoplasia	77
Dysplasia	
LGD	8
HGD	1
UC-associated cancer	7

B, TNM stage	

Stage	Histological differentiation

T1N0M0 with HGD	Poor
T3N0M0 with HGD	Well
T3N0M0 with LGD	Poor
T3N0M0 with HGD	Moderate
T3N0M0 with LGD	Well
T4N0M0 with LGD	Moderate
T4N0M0 with HGD	Well

UC, ulcerative colitis; LGD, low-grade dysplasia; HGD, high-grade dysplasia; TNM, tumor node metastasis.

**Table II:** Characteristics of patients with and without UC-associated neoplasia.

	Non-neoplasia, n=77	UC-associated neoplasia, n=16	P-value
Gender, n (male/female)	39/38	10/6	0.4235
Age at UC diagnosis, years (range)	29 (17–59)	27 (17–55)	0.6357
Extent of disease, n (%)			
Pancolitis	63 (82)	11 (69)	0.3056
Left-sided colitis	14 (18)	5 (31)	
Duration of disease, years (range)	6 (1–28)	11 (1–28)	0.0172

UC, ulcerative colitis.

**Table III:** Multivariate analysis of the utility of disease duration and high CCL20 expression in the rectal mucosa for predicting the risk of developing UC-associated neoplasia.

Variables	Odds ratio	95% confidence interval	P-value
Duration of disease (<8 years vs. ≥8 years)	4.786	0.071–0.865	0.0287
CCL20 IHC score (low vs. high)	4.786	0.071–0.865	0.0287

UC, ulcerative colitis; IHC, immunohistochemistry.

**Table IV:** Comparison of CCL20 and CCR6 expression in UCAC (n=7) and sporadic cancer (n=15).

Marker	Sporadic colon cancer, n (%)	UCAC, n (%)	P-value
CCL20			
Positive	12 (80)	5 (71)	0.6593
Negative	3 (20)	2 (29)	
CCR6			
Positive	2 (13)	4 (57)	0.0316
Negative	13 (87)	3 (43)	

UCAC, ulcerative colitis-associated cancer.

## References

[1] Danese S, Fiocchi C (2011). Ulcerative colitis. N Engl J Med.

[2] Eaden JA, Abrams KR, Mayberry JF (2001). The risk of colorectal cancer in ulcerative colitis: a meta-analysis. Gut.

[3] Cho JH, Brant SR (2011). Recent insights into the genetics of inflammatory bowel disease. Gastroenterology.

[4] Thompson AI, Lees CW (2011). Genetics of ulcerative colitis. Inflamm Bowel Dis.

[5] Slaughter DP, Southwick HW, Smejkal W (1953). Field cancerization in oral stratified squamous epithelium; clinical implications of multicentric origin. Cancer.

[6] Chai H, Brown RE (2009). Field effect in cancer-an update. Ann Clin Lab Sci.

[7] Zimmerman NP, Vongsa RA, Wendt MK, Dwinell MB (2008). Chemokines and chemokine receptors in mucosal homeostasis at the intestinal epithelial barrier in inflammatory bowel disease. Inflamm Bowel Dis.

[8] Koelink PJ, Overbeek SA, Braber S (2012). Targeting chemokine receptors in chronic inflammatory diseases: an extensive review. Pharmacol Ther.

[9] Williams IR (2006). CCR6 and CCL20: partners in intestinal immunity and lymphorganogenesis. Ann NY Acad Sci.

[10] Izadpanah A, Dwinell MB, Eckmann L (2001). Regulated MIP-3alpha/CCL20 production by human intestinal epithelium: mechanism for modulating mucosal immunity. Am J Physiol Gastrointest Liver Physiol.

[11] Kwon JH, Keates S, Bassani L (2002). Colonic epithelial cells are a major site of macrophage inflammatory protein 3alpha (MIP-3alpha) production in normal colon and inflammatory bowel disease. Gut.

[12] Kaser A, Ludwiczek O, Holzmann S (2004). Increased expression of CCL20 in human inflammatory bowel disease. J Clin Immunol.

[13] Katchar K, Kelly CP, Keates S (2007). MIP-3alpha neutralizing monoclonal antibody protects against TNBS-induced colonic injury and inflammation in mice. Am J Physiol Gastrointest Liver Physiol.

[14] Cook DN, Prosser DM, Forster R (2000). CCR6 mediates dendritic cell localization, lymphocyte homeostasis, and immune responses in mucosal tissue. Immunity.

[15] Varona R, Cadenas V, Flores J (2003). CCR6 has a non-redundant role in the development of inflammatory bowel disease. Eur J Immunol.

[16] Wong SC, Lo SF, Lee KC (2002). Expression of frizzled-related protein and Wnt-signalling molecules in invasive human breast tumours. J Pathol.

[17] Itzkowitz SH, Present DH, Crohn’s and Colitis Foundation of America Colon Cancer in IBD Study Group (2005). Consensus conference: Colorectal cancer screening and surveillance in inflammatory bowel disease. Inflamm Bowel Dis.

[18] Eaden JA, Mayberry JF, British Society for Gastroenterology; Association of Coloproctology for Great Britain and Ireland (2002). Guidelines for screening and surveillance of asymptomatic colorectal cancer in patients with inflammatory bowel disease. Gut.

[19] Ullman T, Odze R, Farraye FA (2009). Diagnosis and management of dysplasia in patients with ulcerative colitis and Crohn’s disease of the colon. Inflamm Bowel Dis.

[20] Velayos FS, Liu L, Lewis JD (2010). Prevalence of colorectal cancer surveillance for ulcerative colitis in an integrated health care delivery system. Gastroenterology.

[21] Chen R, Rabinovitch PS, Crispin DA (2003). DNA fingerprinting abnormalities can distinguish ulcerative colitis patients with dysplasia and cancer from those who are dysplasia/cancer-free. Am J Pathol.

[22] Neumann H, Vieth M, Langner C (2011). Cancer risk in IBD: how to diagnose and how to manage DALM and ALM. World J Gastroenterol.

[23] Matsumoto T, Kudo T, Jo Y (2007). Magnifying colonoscopy with narrow band imaging system for the diagnosis of dysplasia in ulcerative colitis: a pilot study. Gastrointest Endosc.

[24] Neumann H, Kiesslich R, Wallace MB, Neurath MF (2010). Confocal laser endomicroscopy: technical advances and clinical applications. Gastroenterology.

[25] Kiesslich R (2010). Chromoendoscopy: what is its true value for ulcerative colitis surveillance?. Dig Dis.

[26] Bista RK, Brentnall TA, Bronner MP (2011). Using optical markers of nondysplastic rectal epithelial cells to identify patients with ulcerative colitis-associated neoplasia. Inflamm Bowel Dis.

[27] Ghadjar P, Rubie C, Aebersold DM, Keilholz U (2009). The chemokine CCL20 and its receptor CCR6 in human malignancy with focus on colorectal cancer. Int J Cancer.

[28] Brand S, Olszak T, Beigel F (2006). Cell differentiation dependent expressed CCR6 mediates ERK-1/2, SAPK/JNK, and Akt signaling resulting in proliferation and migration of colorectal cancer cells. J Cell Biochem.

[29] Rubie C, Oliveira V, Kempf K (2006). Involvement of chemokine receptor CCR6 in colorectal cancer metastasis. Tumour Biol.

[30] Connell WR, Talbot IC, Harpaz N (1994). Clinicopathological characteristics of colorectal carcinoma complicating ulcerative colitis. Gut.

[31] Hinton JM (1966). Risk of malignant change in ulcerative colitis. Gut.

[32] Lavery IC, Chiulli RA, Jagelman DG (1982). Survival with carcinoma arising in mucosal ulcerative colitis. Ann Surg.

[33] Mikami T, Yoshida T, Numata Y (2011). Invasive behavior of ulcerative colitis-associated carcinoma is related to reduced expression of CD44 extracellular domain: comparison with sporadic colon carcinoma. Diagn Pathol.

